# Preference, Perception, and Acceptability of Fluid Gels as a Potential Age-Appropriate Dosage Form for Elderly Patients with Dysphagia

**DOI:** 10.3390/gels8040218

**Published:** 2022-04-01

**Authors:** Zul Hadif Abd Aziz, Haliza Katas, Marhanis Salihah Omar, Noraida Mohamed Shah, Salma Mohamad Yusop, Mohamad Nasir Shafiee, Siti Fatimah Mohd Tarmizi

**Affiliations:** 1 Centre for Drug Delivery Technology, Faculty of Pharmacy, Universiti Kebangsaan Malaysia, Kuala Lumpur 50300, Malaysia; p103519@siswa.ukm.edu.my; 2 Centre for Quality Management of Medicines, Faculty of Pharmacy, Universiti Kebangsaan Malaysia, Kuala Lumpur 59300, Malaysia; marhanis@ukm.edu.my (M.S.O.); noraida_mshah@ukm.edu.my (N.M.S.); 3 Department of Food Sciences, Faculty of Science and Technology, Universiti Kebangsaan Malaysia, Bangi 43600, Malaysia; salma_my@ukm.edu.my (S.M.Y.); sitifatimahmohdtarmizi@gmail.com (S.F.M.T.); 4 Department of Obstetrics and Gynaecology, Universiti Kebangsaan Malaysia Medical Centre, Kuala Lumpur 56000, Malaysia; nasirshafiee@ukm.edu.my

**Keywords:** sheared gel, palatability, swallowability, preference, perception, adherence

## Abstract

The development of pharmaceutical dosage forms that are tailored to specific populations according to their preferences and acceptability could improve medication adherence, which could lead to effective pharmacotherapy. This study evaluated the preference for and perceptions of fluid gels as a potential age-appropriate dosage form for older adults with dysphagia. The palatability and swallowability of the developed fluid gels were also assessed to determine the consumer acceptability of this formulation. A cross-sectional survey was conducted through the electronic distribution of a self-administered questionnaire among adults in Malaysia between April and December 2021. A randomized and double-blinded clinical study was conducted to evaluate the palatability and swallowability of the fluid gels in 30 healthy participants. A cross-sectional study involving 673 respondents revealed that the fluid gels were perceived positively by consumers (64.4%), were easily swallowed (50.8%), were safe to be consumed (45.3%), and were suitable as a new pharmaceutical formulation (43.8%). The clinical study shows that moderately thickened fluid gels masked the bitterness of the medication and were easily swallowed. The newly developed fluid gels were also positively perceived by the participants. Taken together, fluid gels have shown great potential as an innovative oral formulation that is suitable for consumption by elderly patients with dysphagia.

## 1. Introduction

Patient acceptance of medicines is a fundamental aspect in the development of pharmaceutical dosage forms, and it is essential for pharmacotherapy adherence and effectiveness. Patient acceptability is defined as the willingness and capability of the end users and their caregivers to administer and use the medicine as intended [1]. It is imperative to achieving successful therapeutic outcomes [2], and the prescription of patient-preferred formulations may lead to better treatment adherence [3,4]. Several elements contribute to the acceptability of oral medicine, including the palatability and ease of swallowing, as well as the patient preference and perception toward the medicine. The palatability of oral pharmaceutical dosage forms is affected mainly by taste, texture, and mouthfeel, whereas the ease of swallowing is affected by both the medicinal product design and the patient’s physiological and/or psychological ability to swallow [3,5]. In previous studies, patients have shown their preferences and expectations toward a particular oral dosage form. For example, capsules were found to be the most preferred and were perceived to be easier to swallow, safe, and very effective [2]. Patient preferences for oral solid formulations were also influenced by the appearance, the number of units per administration, and the number of administrations per day [4]. A liquid formulation with flavor was positively perceived by older populations. However, different groups perceived it differently. For instance, an unflavored liquid formulation was positively received by elderly men but negatively received by elderly women [6].

Liquid formulations are crucial for specific populations, such as pediatrics, geriatrics, and patients with dysphagia who commonly have difficulties in swallowing, as they are easy to swallow and offer a flexible dosage [7]. However, conventional liquid formulations are suboptimal alternatives to solid medications, and they have low acceptability in elderly patients with dysphagia [3,6]. An alternative to conventional oral liquid medication is necessary in order to overcome these conditions, which include formulation instability, unpleasant taste, and aspiration risk in patients with dysphagia [8,9].

Dysphagia is a major geriatric syndrome with high prevalence among elderly patients. Owing to the difficulty in swallowing, older patients with dysphagia require an appropriate oral dosage form, or a modification of the dosage form, for safer administration of the oral medication. In general, increasing the bolus viscosity is widely practiced as a strategy for the management of dysphagia [10,11]. High-viscosity liquids are frequently recommended to reduce the risk of airway inversion and to increase the swallowing safety by reducing the bolus velocity [12]. Recently, fluid gels with different viscosities have been developed by Abd Aziz et al. (2021) as potential age-appropriate dosage forms for patients with dysphagia. The fluid gels developed have good physicochemical properties, with a nectar and honey consistency and a similar dissolution profile to marketed suspension [13].

A fluid gel is a suspension of microgel particles that is produced by applying shear force during the gelation of a biopolymer solution [14]. Fluid gels have been shown to be promising drug delivery formulations and are suitable as thickened liquids to deliver medication in patients with dysphagia [13,15]. The higher viscosity of fluid gels provides an advantage in swallowing, taste masking, and stability, and it reduces the risk of aspiration or choking [12,15,16]. Thus, this study assessed the preference and rejection factors of liquid formulations, and it provides a preliminary insight into the palatability and swallowability of oral fluid gels as pharmaceutical formulations. This knowledge is important for predicting the desired properties of fluid gel formulations for their administration to targeted populations, such as pediatrics, geriatrics, and patients with dysphagia, who will benefit most from this formulation.

## 2. Results

### 2.1. Public Preferences and Perceptions toward Fluid Gels

#### 2.1.1. Demographics

In total, 673 participants completed the questionnaire. The participants included 324 men (48.1%), with a mean (±SD) age of 36.2 (±12.7) years. A total of 437 respondents were working, while the remaining 236 were unemployed (students, pensioners, or housewives), with 31.2% (n = 210) of the respondents having monthly incomes of more than RM 5001 (USD 1186.76). The demographic information of the respondents is presented in Table 1.

#### 2.1.2. Preferences of Oral Liquid Formulations for Medicines

**Figure 1:**
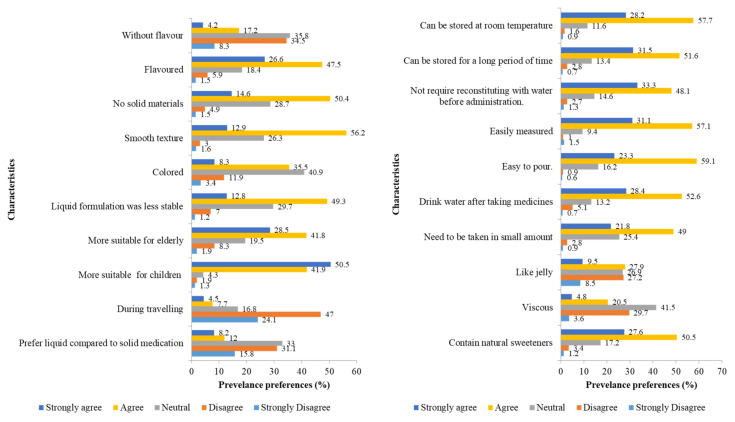
Preferences of oral liquid formulations for medicines, Section 2 (n = 673).

Flavored liquid formulations (74.1%) with smooth textures (69.1%) and an absence of solid materials (65%) were the preferred formulations. Natural flavors, such as bee honey, were preferred over synthetic (78.1%) flavors. The formulations were also preferred if reconstitution was not required (81.4%) and if they could be taken in small doses (70.8%). However, almost half of the respondents preferred to take solid medicines, such as tablets or capsules, compared to liquid medicines (46.9%), particularly while travelling (71.1%). The preferred characteristics of the liquid formulation are shown in Figure 1. Overall, the mean score (±SD) of positive preferences toward the liquid formulation was 71.6 ± 0.3%, regardless of the social demography. However, there was no significant difference between age groups (*p* = 0.949), gender (*p* = 0.253), occupation (*p* = 0.246), and monthly income (*p* = 0.417) in terms of the preference toward liquid formulations. The preferences, according to the respondents’ social demography, are listed in Table 2.

#### 2.1.3. Factors That Influence Rejection of Oral Liquid Formulations for Medicine

Most of the respondents did not have any difficulties or problems in administering liquid oral medications. The rejection factors for liquid formulations are listed in Table 3. The frequent problems faced by the respondents with regard to liquid formulations were difficulties in identifying the medicine after the removal of the packaging (54.7%) and storing the medicine for a long period of time (44.0%) because of its instability. The participants also indicated discomfort in taking oral medicines with unpleasant tastes (42.3%) or containing small particles (41.8%). The mean score (±SD) of the negative perception toward liquid formulations was only 27.7% ± 21.1%. Negative perceptions toward liquid formulations were significantly higher in women (30.0% ± 35.0%; *p* < 0.001) in the age group >45 years (30.0% ± 35.0%; *p* < 0.001), pensioners (35.0% ± 37.5%; *p* < 0.001), and people with monthly incomes of more than RM 5001 (30.0% ± 30.0%; *p* < 0.001), as shown in Table 2.

#### 2.1.4. Perceived Benefits and Risks of Fluid Gels

**Figure 2:**
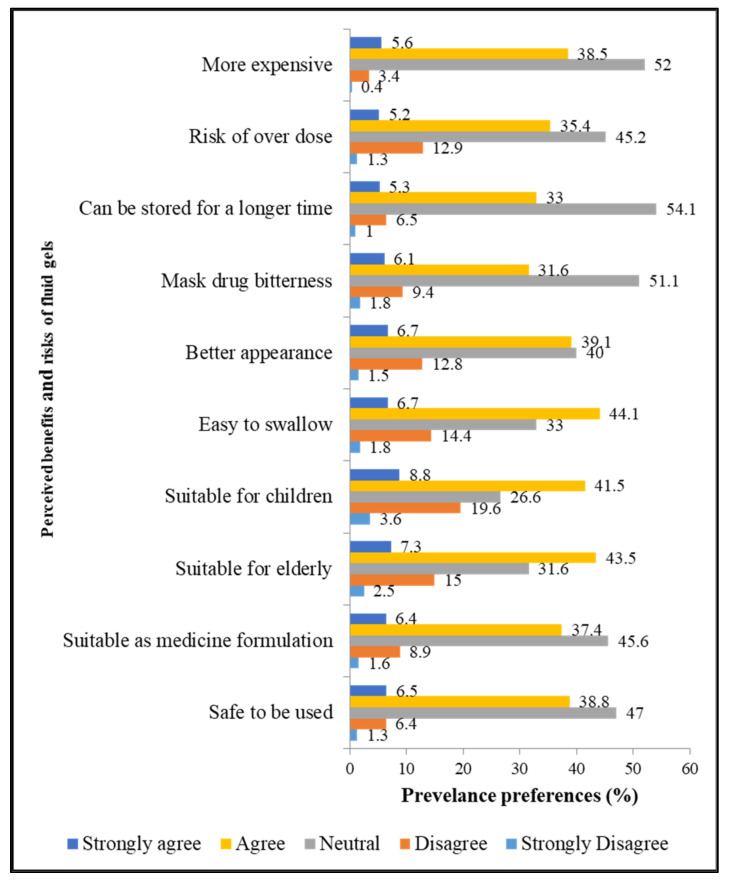
Perceived benefits and risks of fluid gels, Section 4 (n = 673).

The perceived benefits and risks of fluid gels are illustrated in Figure 2. The participants believed that fluid gels could be easily swallowed (50.8%), safely consumed (45.3%), and could mask drug bitterness (37.7%). A high percentage of respondents also had a positive perception that the fluid gels are suitable as a medicine or pharmaceutical formulation (43.8%), and that they are suitable for use in pediatric (50.3%) and geriatric (50.8%) populations. Overall, the mean score (±SD) for the perceived benefits was 64.4% ± 9.7%. The perceived benefits of fluid gels were significantly higher among the age group > 45 years (66.0% ± 12.0%; *p* = 0.006) and self-employed individuals (66.0% ± 13.0%; *p* = 0.024).

#### 2.1.5. Relationship between Preference and Negative Perception toward Liquid Formulations and Perceived Benefits of Fluid Gels

There was a significant negative and very weak correlation between the subject’s preference for liquid formulation and the subject’s negative perception (rejection) toward liquid formulation (r = −0.089, *p* = 0.021). Moreover, there was a significant positive and weak correlation between the subject’s preference for liquid formulation and the subject’s perceived benefit of fluid gels (r = 0.258, *p* < 0.001), and a nonsignificant negative and very weak correlation between the subject’s negative perception and the perceived benefit of fluid gels (r = −0.007, *p* = 0.856).

### 2.2. Sensory Evaluation Studies

#### 2.2.1. Palatability of Fluid Gels

**Figure 3:**
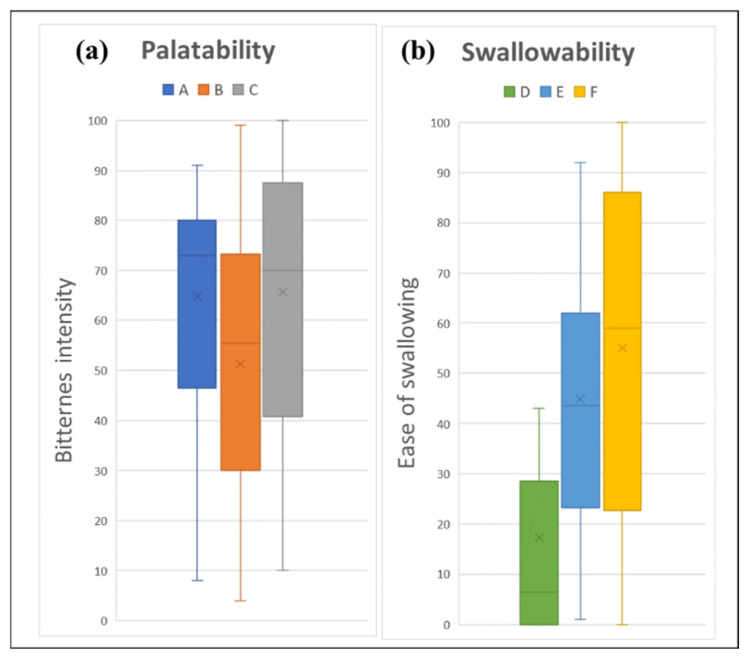
Sensory assessment of fluid gel: (**a**) palatability assessment; and (**b**) swallowability assessment, where 0 is “None” and 100 is “Strong bitterness” for palatability assessment, and 0 is “Very easy” and 100 is “Very difficult” for swallowability assessment. Fluid gels formulated with different consistency were assigned as samples A and D (thin liquid), B and E (mildly thickened liquid), and C and F (highly thickened liquid).

A thicker fluid gel of 0.5% gellan gum (VAS score = 51.23) was able to mask the intensity of medication bitterness better than a thinner fluid gel of 0% gellan gum (VAS score = 64.73) (Figure 3a). However, a further increase in the gellan gum concentration to 1% cancelled the taste-masking effect of the fluid gel (VAS score = 65.66). Friedman’s ANOVA showed significant differences between all samples (*p* < 0.001), and Wilcoxon’s signed rank test comparing each pair showed a significant difference between Samples A and B (*p* < 0.001) and Samples B and C (*p* = 0.013).

#### 2.2.2. Swallowability of Fluid Gels

All of the fluid gels were easy to swallow, with mean VAS scores of 17.27, 44.87, and 55.03 for Samples D, E, and F, respectively (Figure 3b). A thinner fluid gel was significantly easier to swallow by the healthy volunteers than a thicker fluid gel (*p* < 0.001). Wilcoxon’s signed rank test comparing each pair showed a significant difference between Samples D and E and Samples D and F, both with *p* < 0.001. However, no significant difference was observed between the thickened fluid gel (Samples E and F (*p* = 0.106)).

**Figure 4:**
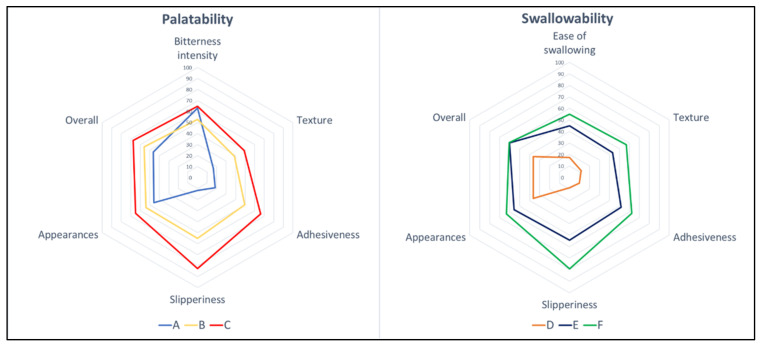
Radar charts for bitterness intensity/ease of swallowing, texture, adhesiveness, slipperiness, appearance, and overall, for fluid gels. Each sensory item is described by its population mean for the 100 mm visual analogue scale (VAS), where 0 is the lowest possible intensity of the stimulus, and 100 is the highest.

**Figure 5:**
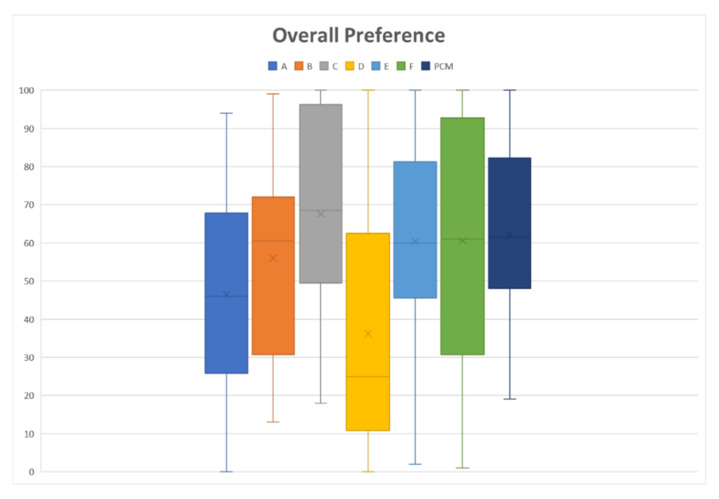
Overall preference toward fluid gel formulation, where 0 is “Extremely like” and 100 is “Extremely dislike”. Paracetamol (PCM) acts as a control, which is a perception when taking marketed paracetamol suspension/syrup. Fluid gels formulated with different consistency were assigned as samples A and D (thin liquid), B and E (mildly thickened liquid), and C and F (highly thickened liquid).

The results of the sensory assessment are summarized in Figure 4. The radar chart shows the mean results for all six sensory attributes that were evaluated: bitterness intensity/ease of swallowing, texture, adhesiveness, slipperiness, appearance, and overall likeness. In all attributes, the thin liquid had a low score for most properties, which indicates a better acceptance than the thickened liquid. For the overall preference toward fluid gels, the thinner fluid gel (VAS score of 46.53 for Sample A, and 36.23 for Sample D) had a greater preference than that of the control (VAS score = 62.10), whereas both thickened liquids had the same preference as that of the control, with VAS scores in the range of 55.97 to 67.53 (*p* < 0.001), as shown in Figure 5.

## 3. Discussion

Novel pharmaceutical formulations that minimize toxicity and improve drug efficacy offer many benefits to consumers and new avenues for pharmaceutical companies. As patients are the end users of medicines, the prescribers and pharmaceutical industries need to understand their preferences and their acceptability of medicines in order to nurture adherence to cost-effective therapies. Adherence to pharmaceutical formulations is affected by multidimensional aspects, including the palatability and swallowability of the formulation, as well as consumer preferences and perceptions toward the formulation. This study explored the preference toward and acceptability of a novel fluid gel so that it can be used as an alternative formulation to deliver medicine effectively and safely, particularly in the younger generation, the elderly, and in patients with dysphagia.

The fluid gels were perceived to be easily swallowed and safe for consumption. This finding is in agreement with that of previous studies that report that a fluid with sufficient viscosity can be easily swallowed without causing any discomfort [12,16,17]. A formulation with suitable mechanical properties for specific populations can be easily produced with fluid gels by controlling the formulation parameters [13,15]. However, liquid medicines, which are easy to measure and pour, were preferred by the general population. Similar to other liquid medications, fluid gels can be easily measured and poured, which offers dosage flexibility and can be an advantage in the elderly population. Extra care is required in designing medicinal products for elderly patients, as they face several complexities in self-administered medicines because of their impaired cognition and reduced physical capabilities [1,7]. Formulations that are easy to measure, pour, and swallow are important for fostering adherence among these populations.

This study discovered that flavored medications were preferred by consumers, which is in agreement with other studies [6,18]. Medicines without added flavors are naturally bitter, unpleasant, and unpalatable, which hinders treatment completion. Flavoring is added to liquid formulations to reduce the aversive taste of the corresponding drug. Natural flavors, such as bee honey or mint, can be added to liquid formulations, as they were found to be preferred by the consumers in this study. Natural flavors are preferred, which is due to the consumers’ perception that they are healthier and safer than artificial flavoring agents [19]. The gels designed in this study were tailored to elderly patients, such that a nondiabetic sweetener and natural flavor were used to provide a medium sweetness that would be acceptable to the elderly during long-term treatment.

Unlike solid dosage forms, masking the bitterness of liquid formulations is difficult and leads to a high rejection rate among patients [7]. Masking the aversive taste of liquid medications can also be achieved by increasing the liquid viscosity by using a thickening agent. The palatability assessment showed that mildly thickened liquids were able to mask the medicine’s bitterness, as compared to thin liquids, though the masking effect was nullified in the highly thickened liquids. Taste masking is achieved primarily through the prevention of the direct contact of the drug substances that are dissolved in the buccal cavity with the taste buds on the tongue [20]. However, overly thickened liquids will increase the contact as the oral transit time is prolonged, and this increases the risk of post-swallow residue in the mouth [21,22], which cancels the taste-masking effect, as is shown by highly thickened liquids.

This study found that all the fluid gels were easily swallowed, probably because of the smooth texture of the formulation. Thin liquids are the easiest to swallow by healthy individuals, compared to thickened liquids, as they require less effort in swallowing [22,23]. Thin liquids are perceived of as less viscous, less adhesive, and easier to maneuver in the mouth than thicker fluids [23]. The rheological properties of the formulation are important as they affect the palatability, and the organoleptic qualities are often reported to worsen with an increase in the consistency of the fluid [10,23]. Thicker liquids have been shown to have a detrimental impact on the mouthfeel, as greater effort is needed by the throat muscles to pass the liquid through the oral cavity and swallow [17,23]. Nevertheless, thicker liquids are known to exhibit prolonged oral transit times compared with thinner liquids, which can be used in the management of patients with dysphagia [21]. Thickened liquids can be safely used in the administration of medicine to geriatric patients with dysphagia to prevent suffocation and choking [22]. Therefore, oral fluid gels could be a good alternative to traditional liquid forms, such as syrups, suspensions, or drinks.

The thickened fluid gels also had a low positive overall likeness as compared to the thin fluid gels among the healthy volunteers in this study. Low positive ratings toward thickened fluid gels may result from neophobia, which is a predisposition to reject formulations from a lack of experience with new products [24,25]. Variation in the perception of the novel dosage form over time, particularly in terms of acceptability, can be explored by a future reintroduction of the formulation to the same participants. Furthermore, as different subsets of the population may be expected to have disparate sensory appreciations [17], the opinions of healthy adults with regard to the sensory qualities may not be applicable to the elderly and to patients with swallowing difficulties. This study was conducted on healthy volunteers under the supervision of the research team and a controlled environment. Thus, this study may help to understand fluid gel suitability and acceptability without definitive conclusions about their safety and acceptability when administered by lay people or ill elderly people in their homes. The study should be extended to investigate the acceptability of these dosage forms in geriatrics, patients with swallowing difficulties, and in those who could benefit the most from these formulations.

A majority of the respondents agreed that fluid gels and liquid formulations are more suitable for the young and the elderly than solid medicines, mainly because these groups cannot easily swallow solid medicines [3]. Liquid formulations provide maximal dosing flexibility and precise and individualized doses to young and elderly populations [7,26]. In cases of difficulties in swallowing the solid dosage form, liquid formulations should be considered first in these populations, as altering a medication dosage form may lead to the unlicensed use of the medicine, and can potentially alter the bioavailability, toxicity, and stability of the medicine. The involvement of a caregiver is common in both populations, and the ability and willingness of the caregiver to administer a medicine determines the acceptability of the medicine, as well as the outcome of the treatment [3]. These studies found that fluid gel formulations were easy to administer to these populations. Semisolid preparations, such as oral fluid gels, can overcome several disadvantages of conventional oral liquid preparations, as they are generally safer to swallow, can be administered in small quantities, and are easier to handle and transport [1,23]. Fluid gel is a potentially superior liquid formulation that delivers safer medications for all populations, including patients with dysphagia. Therefore, the development of a cost-effective pharmacotherapy formulation that is easy to handle and administer may increase the proclivity of patients for medicinal adherence.

## 4. Study Limitations

This study has a few limitations. First, we recruited participants who self-assessed as healthy and who were able to self-administer online questionnaires, which excluded dysphagic patients or people diagnosed with swallowing difficulties. Therefore, studies that focus on these populations should be conducted to further investigate their acceptance toward fluid gels. Second, the perceptions of the respondents from an online questionnaire toward fluid gels were merely based on presumption, as most of the respondents had never had any experience in consuming a fluid gel. However, a clear illustration of the fluid gel was provided before the respondents answered the survey. This study provides preliminary data on fluid gels, upon which further investigation should be conducted. Further research is needed to formulate fluid gels in a pharmaceutical dosage form and to determine the ease of swallowing by patients to ensure their safety. The findings of this study strongly suggest that the pharmaceutical industry should consider manufacturing liquid gel formulations, as they are preferred by consumers and could benefit young children, the elderly, and patients with dysphagia.

## 5. Conclusions

In summary, fluid gels were perceived positively by the healthy volunteers and the general population, and they can be formulated as an oral dosage form. Several formulation characteristics are important to increase the consumers’ preferences for liquid dosage forms, such as pleasant flavor, and ease of swallowing and measurement. Fluid gel that was thickened to a medium consistency (0.5% gellan gum) was able to mask medicine bitterness and could be swallowed easily. Thus, a novel pharmaceutical formulation that addresses problems that are associated with specific populations, and that is formulated with the most preferred characteristics, is expected to increase consumer medication acceptability and adherence, which is highly vital in elderly patients that are dependent on their pharmacotherapy.

## 6. Materials and Methods

A cross-sectional survey and a clinical trial were conducted to enhance patient adherence by comprehending their preferences toward and acceptability of fluid gels. The survey assessed the patients’ preferences, the rejection factors, and the perceived benefits and risks with regard to fluid gels. A clinical study was conducted by evaluating the palatability and swallowability of fluid gels in 30 healthy participants. All procedures performed in studies involving human participants were in accordance with the ethical standards of the institutional and/or national research committee (Research Ethics Committee, the Universiti Kebangsaan Malaysia Medical Center (UKM) (PPI/111/8/JEP-2021-596), and with the 1964 Helsinki declaration and its later amendments or comparable ethical standards.

### 6.1. Public Perception and Preference Assessment

A cross-sectional survey was conducted through the electronic distribution of a self-administered questionnaire to the general adult population in Malaysia between April and December 2021, through an online survey form that used a five-point Likert scale. Social media networks were used to obtain suitable responses. Those who did not complete the survey were excluded from the study. 

The first section of the questionnaire consisted of the respondents’ demographic information, including age, sex, ethnicity, occupation, and monthly income. The second section outlined their preferences toward two oral liquid medicines (conventional and novel fluid gel formulations). The definitions of the conventional and novel fluid gel formulations were provided at the beginning of the questionnaire. All key pharmaceutical characteristics of an ideal oral formulation, such as the texture, stability, flavor, administration, and storage, were included and were based on a five-point Likert scale (from 1 = strongly disagree to 5 = strongly agree). Questions that were negatively worded were reverse scored before summing the scores for each question. High scores signify positive preferences for liquid formulations, whereas low scores indicate negative preferences. 

The third section of the questionnaire focused on the factors that influence the preferences toward liquid formulations for medicine, using yes or no answers. Finally, the fourth section of the questionnaire asked about the perceived benefits and risks of the newly developed fluid gels on the basis of a five-point Likert scale (from 1 = strongly disagree to 5 = strongly agree). The scores were taken from the sum of the answers to all the items. As all the statements were positively worded, except for the risk-related questions (9 and 10 (reversed scale)), low scores signified the negatively perceived benefits and risks of the fluid gel, whereas high scores indicated the positively perceived benefits and risks. All questions were developed and/or adapted from previous studies [3,6,8].

The development, pretesting, and piloting of the research questionnaire was accomplished on the basis of the principles of patient and public involvement (PPI) in research. A small-scale informal pilot of the questionnaire was conducted with 10 participants to ensure the face validity, to assess the comprehension of the questionnaire, and to improve the study by considering the feedback of the respondents. Following the pilot phase, minor changes were made to the questionnaire to improve the clarity. The total Cronbach’s alpha score was in the range of 0.963–0.988, which indicates good reliability and internal consistency. 

### 6.2. Sensory Assessment Study

#### 6.2.1. Materials

Paracetamol, low-acetylated gellan gum (Gelzan), poly(ethylene glycol), and sodium citrate were purchased from Sigma-Aldrich (Petaling Jaya, Malaysia). Propylene glycol and citric acid were obtained from Nacalai Tesque, Inc., Kyoto, Japan. Methyl paraben (sc-218815) was obtained from Santa Cruz Biotechnology. Food-grade xylitol powder (87-99-0, Take It Global Sdn. Bhd.), honey powder (A&T Ingredients Sdn. Bhd.), and sucralose (56038-13-2, Take It Global Sdn. Bhd.) were purchased from local suppliers.

#### 6.2.2. Preparation of Administration Media

The fluid gels were prepared in a good manufacturing practice (GMP)-compliant facility for food processing (*Fasiliti Loji Pandu Makanan*), at the Faculty of Science and Technology, the Universiti Kebangsaan Malaysia. Fluid gels with viscosities suitable for different levels of dysphagia, as recommended by the International Dysphagia Diet Standardization Initiative [27], were prepared. Fluid gels were formulated for Level 1 (thin), Level 2 (mildly thickened liquid), and Level 3 (highly thickened liquid), and assigned as Samples A and D (Level 1), B and E (Level 2), and C and F (Level 3). Briefly, a jacketed vessel with a magnetic stirrer attached to a water bath was used to prepare the fluid gels, based on our previous study [13]. Table 4 lists the different compositions of each prepared formulation. The beaker filled with 15 mL of distilled water was then heated to 90 °C before adding 5 mL of polyethylene glycol and propylene glycol solution containing low-acyl gellan gum, paracetamol, and methyl paraben. The solution was stirred for 30 min at 90 °C before cooling to 5 °C using a water bath, at a cooling rate of 1–2 °C/min, under stirring at 500 rpm. Sodium citrate and citric acid were added when the temperature of the solution reached 80 °C. Finally, a solution containing xylitol, sucralose, and honey was added when it cooled to 60 °C. The resultant fluid gels were prepacked in a 5 mL container and were stored at 8 °C until further use in our clinical trial.

#### 6.2.3. Study Design

This single-center randomized double-blinded study was conducted at the Hospital Canselor Tuanku Muhriz UKM in Kuala Lumpur, Malaysia, in accordance with the Code of Ethics of the World Medical Association (Declaration of Helsinki). All the participants in this study were recruited through advertisements on social media platforms. Eligible healthy adults aged 18–35 years were included in the study. We excluded from this study individuals with illnesses such as cough and flu; those who were using prescribed medication during the study period; those who had allergies, or the history of an allergy, to any component in the formulations; smokers; pregnant women; and nursing mothers. The sample size was calculated as follows [28]:

n = 2 × (Z_α_ + Z_(1−β)_)^2^ × (α)^2^/Δ^2^

where n is the sample size; and Z is the Z-score, where Z_α_ and Z_1−β_ are the constants set by convention, according to the accepted α error (5%, one-side effect) and the power of the study (80%), respectively. α is the estimated standard deviation, and Δ is the estimated effect size (based on the difference effect of the consistency between the fluid gel and the control, as well as of the taste masking of a similar formulation and its control) [29]. Therefore,

n = 2 × (1.65 + 0.8416)^2^ × 0.5^2^/0.32^2^ n = 30

#### 6.2.4. Sample Assessment

The participants underwent four sessions within a single visit: (1) Training for the bitterness evaluation; (2) Palatability testing; (3) Swallowability testing; and (4) Preference and perception evaluations of fluid gels. The participants were trained in the first session using three different concentrations of the paracetamol solution (0.5, 2.1, and 5.0 mg/mL) in order to standardize the participant bitterness. Participants first tasted 5 mL of each solution by holding it in their mouth for 60 s and spitting it out without swallowing. They were informed of the numerical value and taste description of the solution [30]. 

Taste masking was evaluated in the second session, in which all participants gargled 5 mL of Samples A, B, and C in their mouth for 60 s and then spat it out. For the third session, the participants swallowed 5 mL samples of the blank fluid gels (D, E, and F (without paracetamol)) in their usual manner to evaluate the ease of swallowing. In the sensory evaluation, the participants were blinded to minimize the bias. The participants were assisted by an unblinded assistant who was responsible for the sample preparation. The samples were given in a randomized order to prevent sequential bias. The sample sequence assignment was determined by using blocked randomization (block size = 6) to ensure balance across the study recruitment period [31]. A 10 min interval and a palate cleanser were provided between the sample testing to minimize the participant discomfort and carryover effects. Palate cleansing was performed by drinking room temperature spring water, followed by a piece of lightly salted cracker and room temperature spring water [32]. During the study, the participants were asked to complete a background questionnaire to record demographic information, including sex, age, and health conditions/illnesses. Immediately after testing each sample, the palatability was assessed with the following anchor phrases: bitterness intensity (“None” vs. “Strong bitterness”); texture (“Smooth” vs. “Rough”); adhesiveness (“Does not stick” vs. “Very sticky”); slipperiness (“Slips easily” vs. “Stays in place”); appearance (“Attractive” vs. “Unattractive”); and overall likeness (“Extremely like” vs. “Extremely dislike”), using a 100 mm visual analog scale (VAS). The bitterness intensity was replaced with ease of swallowing (“Very easy” vs. “Very difficult”) for the swallowability assessment [5,25]. The participants were also asked about their perceptions of the overall likeness when taking marketed paracetamol suspension/syrup, based on their past experiences. The participants’ marks on the VAS were transcribed into scores (from 0 to 100), in which the left line of the VAS indicated a positive attribute (0), while the right end line of the VAS (100) indicated a negative attribute. Thus, samples with an average rating of 0–60 were deemed unaversive or palatable. For the last session, the participants were asked structured questions regarding their preferences and perceptions toward fluid gels by using a similar questionnaire from the public perception and preference assessment. The in vivo sensory study was assessed using the VAS, as it is sensitive in detecting small differences in sensory perception.

#### 6.2.5. Data Analysis

Data were entered and analyzed using the Statistical Package for Social Science (SPSS) Version 19 (IBM Corp., Armonk, NY, USA). Descriptive results were presented as frequencies, percentages, means (±SD), and medians. Nonparametric tests, such as two-independent sample tests and tests for several independent samples, and statistical analyses were used to determine significant associations between each variable. Correlation, the Mann–Whitney U test, and the Kruskal–Wallis H test were used in the cross-sectional study. Pearson correlations were used to examine the relationships between the preference and negative perception toward liquid formulations, and the perceived benefits of fluid gels.

For the sensory analysis, Friedman’s ANOVA (nonparametric test for related samples) was used to screen for differences between samples, and Wilcoxon’s signed-rank test was used to determine differences between individual sample pairs. A significant level was accepted at a *p*-value less than 0.05 for all analyses, except for the pairwise comparison of the fluid gel samples (*p* < 0.0167 was used, derived from *p* = 0.05 divided by 3 combinations of pairs of sensory analysis).

## Figures and Tables

**Table 1:** Social demographics of consumers (n = 673).

Characteristics	n	%
**Gender**		
Male	324	48.1
Female	349	51.9
**Ethnicity**		
Malay	619	92.0
Chinese	31	4.6
Indian	17	2.5
Others	6	0.9
**Age**		
19–30 years old	280	41.6
31–45 years old	198	29.4
More than 45 years old	208	30.9
**Occupation**		
Working		
Professional	412	61.2
Self-employed	25	3.7
Not working		
Pensioner	25	3.7
Housewife	34	5.1
Student	163	24.2
Unemployed	14	2.1
**Monthly income**		
Less than RM 1000 (USD 237.30)	201	29.9
RM 1001 (USD 237.54)–RM 3000 (USD 711.91)	151	22.4
RM3001 (USD 712.15)–RM 5000 (USD 1186.52)	111	16.5
More than RM 5001 (USD 1186.76)	210	31.2

RM 1 is equal to USD 0.24, converted using Google Finance on 23 March 2022.

**Table 2:** Mean scores of preferences toward liquid formulations, negative perception toward liquid formulations, and perceived benefits of fluid gels, based on demographic data (n = 673).

Characteristics	Preferences toward Oral Liquid Formulation		Negative Perception toward Liquid Formulation		Perceived Benefits of Fluid Gels	
	Median ± IQR	*p* Value	Median ± IQR	*p* Value	Median ± IQR	*p* Value
**Gender**						
Male (n = 324)	71.0 ± 8.0	0.253	20.0 ± 25.0	<0.001 **	64.0 ± 12.0	0.594
Female (n = 349)	72.0 ± 8.5		30.0 ± 35.0		64.0 ± 12.0	
**Age groups (years)**						
19–30 (n = 280)	71.5 ± 9.0	0.949	20.0 ± 25.0	<0.001 **	64.0 ± 10.0	0.006 *
31–45 (n = 198)	71.0 ± 8.0		25.0 ± 35.0		62.0 ± 12.5	
More than 45 (n = 208)	71.0 ± 7.0		30.0 ± 35.0		66.0 ± 12.0	
**Occupation**						
Professional (n = 412)	71.0 ± 8.0	0.246	25.0 ± 30.0	<0.001 **	64.0 ± 12.0	0.024 *
Self-employed (n = 25)	72.0 ± 8.5		15.0 ± 35.0		66.0 ± 13.0	
Pensioner (n = 25)	71.0 ± 6.0		35.0 ± 37.5		62.0 ± 18.0	
Housewife (n = 34)	70.5 ± 9.0		27.5 ± 47.5		60.0 ± 10.0	
Student (n = 163)	71.0 ± 9.0		15.0 ± 25.0		66.0 ± 12.0	
Not working (n = 14)	69.0 ± 7.75		25.0 ± 46.3		64.0 ± 8.5	
**Monthly income (RM)**						
Less than RM 1000 (USD 237.30) (n = 201)	71.0 ± 9.0	0.417	20.0 ± 25.0	<0.001 **	66.0 ± 12.0	0.153
RM 1001 (USD 237.54) to RM 3000 (USD 711.91) (n = 151)	71.0 ± 8.0		25.0 ± 35.0		62.0 ± 10.0	
RM3001 (USD 712.15) to RM 5000 (USD 1186.52) (n = 111)	72.0 ± 7.0		25.0 ± 35.0		64.0 ± 10.0	
More than RM 5001 (USD 1186.76) (n = 210)	71.0 ± 8.0		30.0 ± 30.0		64.0 ± 12.5	

Mann–Whitney U test (2 groups) and Kruskal–Wallis H test (>2 groups): * significant data (*p* < 0.05); ** significant data (*p* < 0.001). RM 1 is equal to USD 0.24, converted using Google Finance on 23 March 2022.

**Table 3:** Rejection factors with regard to liquid formulations, Section 3 (n = 673).

Factors That May Influence the Preferences of Oral Fluid Formulations for Medicines	Yes		No	
	n	%	n	%
I have problem to understand the instructions for use of medicines because the text is too small.	184	27.3	489	72.7
I have problem to understand the instructions for use of medicines because the information written too difficult to understand.	156	23.2	517	76.8
I have problem to understand the instructions for use of medicines because the information on adverse events is distressing.	169	25.1	504	74.9
I have problem to open my medicines.	50	7.4	623	92.6
I have problem to identify medicines after it has been removed from its packaging.	368	54.7	305	45.3
I have problem to take oral fluid medicines because of the taste.	285	42.3	388	57.7
I have problem to take oral fluid medicines because of their appearance. Example: Sediment or cloudy.	217	32.2	456	67.8
I have problem to take sweetened or flavored oral fluid medicine.	87	12.9	586	87.1
I have problem to take oral medicines containing small particles.	281	41.8	392	58.2
I have problem to swallow solid medicines such as tablets or capsules.	91	13.5	582	86.5
I have problem to swallow medicines. For examples, choking reflex or cough before swallowing.	83	12.3	590	87.7
I have problem with the volume of oral fluid medicines.	161	23.9	512	76.1
I have problem to take frequent oral medicines (more than 2 times a day).	210	31.2	463	68.8
I have problem to pour fluid medicines.	59	8.8	614	91.2
I have problem to measure the right dose before taking it.	154	22.9	519	77.1
I have problem to reconstitute medicines using water.	247	36.7	426	63.3
I have problem to store oral fluid medicines in a suitable condition after it has been removed from its packaging.	264	39.2	409	60.8
I have problem to store oral fluid medicines for a long period of time.	296	44	377	56
I have problem to give oral fluid medicines to young family members (e.g., aged ≤ 12 years old).	252	37.4	421	62.6
I have problem to give oral fluid medicine to elderly family members (e.g., aged ≥ 65 years old).	118	17.5	555	82.5

**Table 4:** Formulations of fluid gel samples.

Ingredients (% *w*/*v* or % *v*/*v*)	Formulations					
	A	B	C	D	E	F
Paracetamol	5	5	5	0	0	0
Gellan gum	0.1	0.5	1.0	0.1	0.5	1.0
Sodium citrate	0.3	0.3	0.3	0.3	0.3	0.3
Citric acid	0.05	0.05	0.05	0.05	0.05	0.05
Methylparaben	0.2	0.2	0.2	0.2	0.2	0.2
Polyethylene glycol	20	20	20	20	20	20
Propylene glycol	20	20	20	20	20	20
Xylose	30	30	30	30	30	30
Sucralose	0.2	0.2	0.2	0.2	0.2	0.2
Honey flavor	2	2	2	2	2	2
Water, up to	100	100	100	100	100	100
